# Effects of Reduced Carbohydrate Intake after Sprint Exercise on Breath Acetone Level

**DOI:** 10.3390/nu13010058

**Published:** 2020-12-27

**Authors:** Naoki Ota, Hiroto Ito, Kazushige Goto

**Affiliations:** Graduate School of Sport and Health Science, Ritsumeikan University, Kusatsu, Shiga 525-8577, Japan; sh0120hp@gmail.com (N.O.); actarmy.jr.riemann2357@gmail.com (H.I.)

**Keywords:** breath acetone, carbohydrate, fat metabolism, substrate utilization, ketone body

## Abstract

Assessment of breath acetone level may be an alternative procedure to evaluate change in fat metabolism. The purpose of the present study was to investigate the effect of insufficient carbohydrate (CHO) intake after sprint exercise on breath acetone level during post-exercise. Nine subjects conducted two trials, consisting of either reduced CHO trial (LOW trial) or normal CHO trial (NOR trial). In each trial, subjects visited to laboratory at 7:30 following an overnight fast to assess baseline breath acetone level. They commenced repeated sprint exercise from 17:00. After exercise, isoenergetic meals with different doses of CHO (LOW trial; 18% for CHO, 27% for protein, 55% for fat, NOR trial; 58% for CHO, 14% for protein, 28% for fat) were served. Breath acetone level was also monitored immediately before and after exercise, 1 h, 3 h, 4 h, and 15 h (on the following morning) after completing exercise. A significant higher breath acetone level was observed in LOW trial than in NOR trial 4 h after completion of exercise (NOR trial; 0.66 ppm, LOW trial; 0.9 ppm). However, breath acetone level did not differ on the following morning between two trials. Therefore, CHO intake following an exhaustive exercise affects breath acetone level during early phase of post-exercise.

## 1. Introduction

Muscle glycogen content has been recognized to be one of critical factors for determining endurance exercise performance [1,2,3,4]. In addition, a single bout of 30 s maximal cycle sprint exercise caused 35% of reduction of muscle glycogen content [5]. Therefore, muscle glycogen content at the onset of the exercise is crucial for maintaining not only endurance exercise but also short-term sprint exercise. On the sport fields, athletes are required to conduct practices or competitions for several times within a day. Therefore, it is important to recover muscle glycogen content after completion of exercise before the start of subsequent exercise. Betts et al. [6] revealed that recovery of muscle glycogen content following an exercise session was accelerated by an adequate quantity (~1 g/body mass/h) carbohydrate CHO intake. In contrast, insufficient CHO intake following an endurance exercise session delayed recovery of muscle glycogen content, while low CHO intake facilitated fat metabolism (e.g., increased plasma-free fatty acid concentration and decreased respiratory exchange ratio) compared with high CHO intake [4,7,8]. Thus, augmented fat metabolism during post-exercise period by insufficient CHO intake would reflect delayed recovery of muscle glycogen content. In addition, it may be associated with impaired endurance exercise performance during subsequent exercise. Therefore, assessment of fat metabolism during the early phase of post-exercise period is valuable.

Generally, fat metabolism can be evaluated using blood variables (e.g., plasma-free fatty acid, serum total ketone concentrations). However, due to an invasive fashion for evaluating these variables, development of non-invasive assessment for fat metabolism is required. Circulating free fatty acid is converted into acetyl-CoA via β-oxidation, and acetyl-CoA is transformed to ketone bodies in the liver. Ketone bodies consist of acetoacetate, 3-hydroxybutanoic acid and acetone. Acetoacetate is initially produced, followed by 3-hydroxybutanoic acid and acetone productions mediated by enzymatic degradation or decarboxylation, respectively [9]. Among ketone bodies, acetone translates to the lung and it is excreted as an exhaled gas due to its small molecular size [10]. Several studies have demonstrated that a positive correlation was observed between breath acetone level and serum beta-hydroxybutyrate concentration [11,12,13,14]. In addition, Anderson [10] suggested that increase in breath acetone level reflected augmented fat metabolism in the liver. Thus, assessment of breath acetone level can be an alternative and non-invasive procedure to evaluate changes in fat metabolism during post-exercise period [15,16]. Additionally, since acetone is produced from free fatty acid (FFA) which is facilitated by insufficient CHO intake [7], reduced CHO intake following an exercise session may rise breath acetone level during post-exercise period. However, no study has been conducted so far to clarify the effect of insufficient CHO intake after completion of an exhaustive exercise session on breath acetone level during post-exercise period. Therefore, the purpose of the present study was to determine the influence of low CHO intake following sprint exercise on breath acetone level. We hypothesized that reduced CHO intake following the sprint exercise would increase breath acetone level.

## 2. Materials and Methods

### 2.1. Subjects

Nine men (mean ± standard error (SE): age, 20.8 ± 0.2 yrs; height, 170.1 ± 1.6 cm; body mass, 65.1 ± 1.8 kg; BMI, 22.7 ± 0.7 kg/m^2^) were recruited. All subjects were healthy, and none of them have participated in any regular training program at the start of the experiment. Subjects were informed of the experimental procedures and possible risks involved in this study. Subsequently, an informed consent was obtained. The present study was approved was approved by the Ethics committee for Human Experiments at Ritsumeikan University, Japan.

### 2.2. Experimental Overview

**Figure 1:**
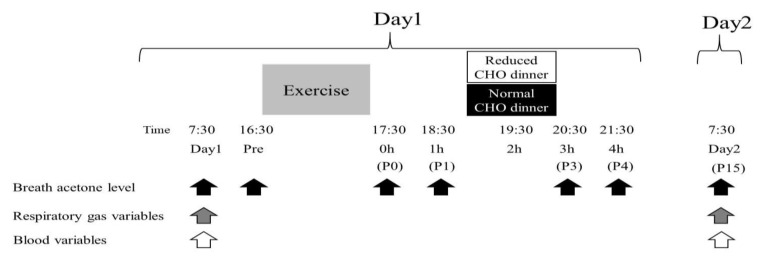
Experimental overview.

All subjects visited the laboratory five times throughout the experiment. On the first visit, they conducted a familiarization session for repeated cycle sprint exercise. From the second to fifth visits, two main trials (consisting of two consecutive days, day1 and day2) were performed. Each main trial consisted of either trial with consuming normal carbohydrate (CHO) meal, following the repeated cycle sprint exercise (NOR trial), or with consuming reduced CHO meal, following the same exercise (LOW trial). These two trials were separated by at least 1 week, and the order of the trials was randomized. In addition, subjects were required to refrain from strenuous physical activity during 24 h prior to main trial on day1. The changes in breath acetone level, respiratory gas, and blood variables were compared until the following morning on day2 (Figure 1).

### 2.3. Familiarization Session

About a week prior to commencing main trials (NOR trial or LOW trial), a familiarization session was performed. Subjects underwent submaximal cycling exercise at 50 W for 5 min. They subsequently conducted 2 × 3 s maximal cycle sprint exercise using an electromagnetically braked cycle ergometer (Power Max III; Konami Corp., Tokyo, Japan). The applied load was equivalent to 5% of body weight for the first cycle sprint exercise and 7.5% of body weight for the second cycle sprint exercise. After completing warm-up exercise, they performed 2 × 30 s maximal cycle sprint exercise using an electromagnetically braked cycle ergometer. The applied load was set at 7.5% of body weight.

### 2.4. Repeated Cycle Sprint Exercise Session on Day1 (Main Trial)

On day1, the subjects came to the laboratory at 7:30, following an overnight fast (from 22:00~). After completing the baseline measurements, the identical meal for breakfast was provided (461 kcal: 77% for CHO, 5% for protein, 19% for fat). In addition, subjects consumed the prescribed lunch (821 kcal: 64% for CHO, 15% for protein, 21% for fat). Any exercise aside from experiment-related exercise was not allowed throughout the day. At 16:30, they returned to the laboratory to start repeated cycle sprint exercise. As a warm-up exercise, subjects initially performed submaximal cycling exercise for 5 min. They subsequently conducted 2 × 3 s maximal cycle sprint exercise using an electromagnetically braked cycle ergometer. The applied load was equivalent to 5% of body weight for the first cycle sprint exercise and 7.5% of body weight for the second cycle sprint exercise.

After completing warm-up exercise, subjects commenced main exercise. The main exercise consisted of 4 × 30 s maximal cycle sprint exercise using an electromagnetically braked cycle ergometer. The applied load was equivalent to 7.5% of body weight. A 10 min of active rest was inserted between the sprints. This exercise protocol has been commonly used as maximal sprint exercise [17]. Subjects were verbally encouraged throughout each sprint to perform the exercise with maximal effort.

### 2.5. Dietary Manipulation in NOR Trial and LOW Trial

On day1, subjects consumed the isocaloric dinner with containing different CHO ratio (from 19:30, 2 h after completing exercise). In NOR trial, the meal was composed of 58% for CHO, 14% for protein, 28% for fat. In contrast, the meal in LOW trial consisted of 18% for CHO, 27% for protein, 55% for fat. The energy intake was matched 1113 kcal between meals. Dinner consisted of normal Japanese food (rice, potato salad, yogurt, Japanese omelet, Japanese fried chicken, tuna, and orange juice). After completing the dinner, the subjects stayed at accommodation located in the university. The sleep duration was unified from 23:00 to 07:00 for all subjects.

### 2.6. Measurements

In the morning on day1 and day2, breath acetone level, blood variables, and resting respiratory gas variables were evaluated, following an overnight fast. Breath acetone level was also evaluated immediately before and after exercise, 1 h, 3 h, 4 h, and 15 h after completing repeated cycle sprint exercise. During the exercise, mean power output and maximal pedaling frequency were recorded. Moreover, heart rate was recorded throughout the exercise using a heart rate monitor (RCX5; POLAR corporation, Kempele, Finland).

#### 2.6.1. Breath Acetone Level

Breath acetone level was evaluated using an automatic breath acetone analyzer (HOSIDEN corporation, Osaka, Japan). Subjects were required to exhale breath into automatic analyzer for 5 s. In each measurement, breath acetone level was evaluated three times. The average value for three measurements was adopted.

#### 2.6.2. Blood Variables

In the morning on day1 and day2, venous blood samples were taken from an antecubital vain. Serum samples were subsequently obtained after a 10-min centrifugation at 4 °C and stored at −80 °C until analysis. From obtained serum samples, serum insulin, acetoacetic acid, 3-hydroxybutanoic acid, and total ketone concentrations were assayed at the clinical laboratory (SRL Inc., Tokyo, Japan). Further blood samples were obtained after each sprint for determinations of blood glucose and lactate concentrations. These concentrations were evaluated using a glucose analyzer (Free Style, Nipro Co., Osaka, Japan) and a lactate analyzer (Lactate Pro2; Arkray Co., Kyoto, Japan), respectively.

#### 2.6.3. Resting Respiratory Gas Variables

In the morning on day 1 and day 2, subjects stayed rest for 10 min at the laboratory. Subsequently, respiratory gas samples were collected for 5 min using a breath-by-breath method and an automatic gas analyzer (AE300S; Minato Medical Science Co., Tokyo, Japan). The collected data were averaged every 30 s to determine respiratory exchange ratio (RER), oxygen consumption (VO_2_), and carbon dioxide output (VCO_2_). Substrate oxidation (fat and carbohydrate oxidation) was evaluated using the equation reported in a previous study [18].

### 2.7. Statistical Analysis

All values are presented as means ± SE. Two-way repeated-measures analysis of variance (two-way ANOVA) was applied to assess the main effect (trial and time) and the interaction (trial × time). When the ANOVA revealed a significant interaction or main effect, the Tukey-Kramer test was performed to identify differences. In addition, paired t-test was used to compare resting respiratory gas and blood variables on day1 and day2. A *p*-value < 0.05 was considered significant for all measurements.

## 3. Results

### 3.1. Power Output and Heart Rate During Repeated Sprint Exercise

Maximal pedaling frequency, mean power output, and relative mean power output (W/kg) significantly decreased during set 3 and set 4 compared with set 1 (*p* < 0.05). However, no significant difference was observed for these variables between two trials (Table 1). Heart rate throughout the exercise session was not different significantly between two trials.

### 3.2. Breath Acetone Level

**Figure 2:**
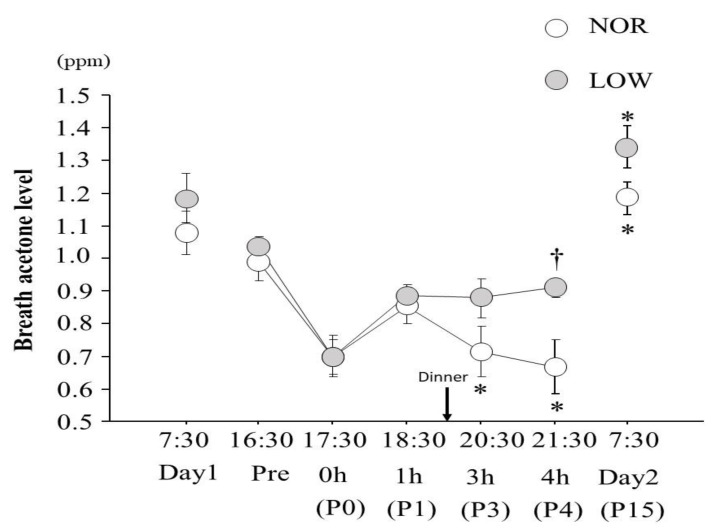
Changes in breath acetone level. Values are means ± SE.* *p* < 0.05 vs. 1 h, † *p* < 0.05 vs. trial.

In NOR trial, breath acetone level was markedly decreased after consuming dinner until 4 h (P4) after exercise. Consequently, a significant reduction was observed at 3 h (P3) and 4 h (P4) compared with 1 h (P1) in NOR trial (*p* < 0.05). At 4 h after exercise, LOW trial showed significantly higher breath acetone level than that in NOR trial (*p* < 0.05). However, there was no significant difference in breath acetone level between trials in the morning on day2 (Figure 2).

### 3.3. Blood Variables

Blood lactate concentration was increased significantly with exercise, but no significant difference was found between the trials. In addition, blood glucose concentration was not different significantly between the two trials (Table 2).

**Figure 3:**
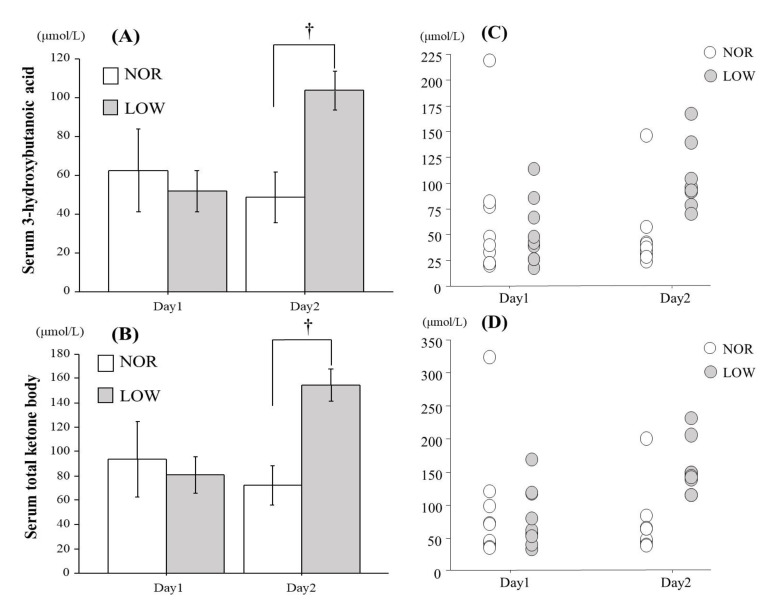
Changes in mean value of serum 3-hydroxybutanoic acid (**A**), total ketone body concentration (**B**), individual value of serum 3-hydroxybutanoi cacid (**C**) and total ketone body concentration (**D**). Values are means *±* SE. † *p* < 0.05 vs. trial.

Figure 3 presents changes in serum 3-hydroxybutanoic acid (A) and total ketone body concentrations (B) in the morning on day1 and day2. On day1, no significant difference was observed between two trials for serum 3-hydroxybutanoic acid or total ketone body concentrations. In contrast, both variables were significantly higher in LOW trial than in NOR trial on day2 (*p* < 0.05). Furthermore, serum acetoacetic acid concentration was significantly higher in LOW trial on day2 (*p* < 0.05). Serum insulin concentration was not different significantly between the trials on day1 and day2.

### 3.4. Resting Respiratory Gas Variables

**Figure 4:**
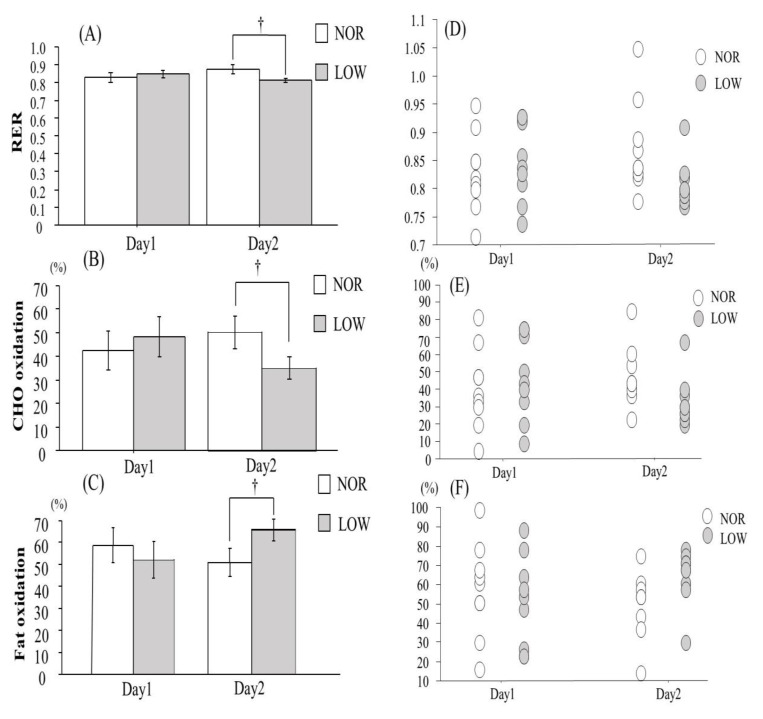
Changes in mean value of RER (**A**), CHO oxidation (**B**), fat oxidation (**C**), individual value ofRER (**D**), CHO oxidation (**E**) and fat oxidation (**F**). Values are means ± SE. † *p* < 0.05 vs. trial.

$\dot{V}$O_2_ and $\dot{V}$CO_2_ did not differ significantly between two trials in the morning on day1 and day2. There was no significant difference in RER between two trials in the morning on day1. However, LOW trial showed significantly lower RER in the morning on day2 (*p* < 0.05). In addition, CHO oxidation was significantly lower in the LOW trial than in NOR trial on day2, whereas LOW trial presented significantly higher fat oxidation (*p* < 0.05, Figure 4).

## 4. Discussion

The present study investigated the effect of CHO intake following an acute sprint exercise session on breath acetone level. The main finding was that breath acetone level following normal CHO intake after the sprint exercise (NOR trial) markedly decreased until 4 h after the completion of exercise. Consequently, LOW trial showed significantly higher breath acetone level compared with NOR trial 4 h after completing the exercise. These findings indicate that breath acetone was strongly affected by CHO intake during early phase of post-exercise.

Mean power output and maximal pedaling frequency during repeated sprint exercise did not differ significantly between the two trials. Additionally, post-exercise blood lactate concentration was not significantly different between the conditions. Therefore, it appeared that sprint exercise in both trials evoked similar physiological stimulus. Catecholamine and growth hormone (GH) promote lipolysis. In addition, exercise-induced elevation of these hormones are highly dependent on exercise intensity [19]. In the present study, since maximal cycle sprint exercise was applied, it appeared that exercise-induced catecholamine and GH elevations were maximized. Moreover, both trials presented comparable increase in breath acetone level 1 h after completing exercise (1 h before consuming the dinner). Therefore, exercise-induced increase in lipolysis would be similar between two trials. In the morning on day2, serum 3-hydroxybutanoic acid and total ketone body concentrations were significantly higher in LOW trial than in NOR trial. Furthermore, fat oxidation at rest on day2 was significantly higher in LOW trial compared with NOR trial, indicating that fat metabolism in the morning on day2 was facilitated in LOW trial. Delayed recovery of muscle glycogen content during post-exercise period might be a reason for augmented fat metabolism in LOW trial. Previous studies showed that recovery of glycogen content following an exhaustive exercise was faster in liver than in muscle [20,21,22]. In addition, reduced CHO intake during post-exercise delayed recovery of muscle glycogen content [4,23]. In addition, insufficient CHO intake led to increase in FFA concentration [4,7]. Although we did not assess glycogen content in either muscle or liver, it is plausible that recovery of muscle glycogen content was delayed due to low CHO intake following sprint exercise on day1. Therefore, reduced CHO intake following sprint exercise augmented fat metabolism the next morning.

Somewhat surprisingly, breath acetone level did not differ significantly between two trials in the morning on day2. Fasted status has shown to increase breath acetone level [14,24]. In the present study, subjects were not allowed to take meals after consuming dinner following the exercise on day1. Therefore, potential difference in breath acetone level between the two trials may be masked due to an overnight fasting. However, it was notable to find that breath acetone level 4 h after completion of exercise (2 h after consuming meal) was significantly higher in LOW trial than in NOR trial. Bovey et al. [25] compared breath acetone level following high CHO (HC trial) and low CHO (LC trial) intake. Consequently, HC trial presented a significant reduction of breath acetone level 1 h after consuming meal. On the other hand, LC trial showed significantly higher breath acetone level compared with HC trial 2 h after consuming the meal. The authors suggested that increase in breath acetone level was abolished by CHO intake.

The present study includes several limitations. Firstly, exercise performance was not evaluated in the morning on day2. Therefore, we are not able to present potential relations between breath acetone level during post-exercise period and recovery of exercise performance. There is a possibility that muscle glycogen content was not recovered to baseline level in LOW trial, which may impair endurance exercise performance. Additionally, since exercise with low muscle glycogen content causes compensatory increase in fat metabolism [26], higher breath acetone level (i.e., augmented fat metabolism) may reflect attenuated exercise performance. Thirdly, we did not evaluate change in serum total ketone concentration after consuming the meal on day1. Thus, relationship between breath acetone level and serum total ketone concentration after consuming the meals has not been clarified. Fourthly, we did not include a fasting trial (without consuming the meal) following exercise. The control trial without consuming the meal will be important in future work to determine breath acetone level in the fasting state following an exhaustive exercise. Finally, the present study utilized repeated cycle sprint exercise. Therefore, further determination of changes in breath acetone level following different types of exercise protocols (e.g., prolonged endurance exercise, team-based sports with 80–90 min of match play) would be informative.

## 5. Conclusions

A significant higher breath acetone level was observed in LOW trial than in NOR trial 4 h after completing exercise. However, breath acetone level did not differ significantly between two trials in the morning on day2. These findings indicate that breath acetone level during early phase of post-exhaustive exercise is affected by CHO intake.

## Figures and Tables

**Table 1:** Exercise performance variables during repeated sprint exercise.

		Set 1	Set 2	Set 3	Set 4
Maximal pedaling frequency (rpm)	NOR	154 ± 3	153 ± 2	148 ± 4 *	144 ± 3 *
	LOW	154 ± 3	148 ± 3	145 ± 2 *	143 ± 2 *
Mean power output (W)	NOR	612 ± 20	583 ± 20	556 ± 21 *	539 ± 18 *
	LOW	585 ± 20	575 ± 18	548 ± 18 *	537 ± 19 *
Relative mean power output (W/KG)	NOR	9.4 ± 0.4	9.0 ± 0.2	8.5 ± 0.2 *	8.3 ± 0.2 *
	LOW	9.0 ± 0.3	8.8 ± 0.2	8.4 ± 0.2 *	8.2 ± 0.2 *

Values are means ± SE.* *p* < 0.05 vs. Set1.

**Table 2:** Blood lactate and glucose concentrations.

		Day1	Pre	Set1	Set2	Set3	Set4	Day2
Lactate (mmol/L)	NOR	1.3 ± 0.1	1.2 ± 0.1	8.1 ± 0.6 *	13.2 ± 0.8 *	17.4 ± 0.8 *	19.8 ± 1 *	1.2 ± 0.1
	LOW	1.4 ± 0.1	1.4 ± 0.1	10.8 ± 0.8 *	13.8 ± 0.5 *	15.6 ± 0.7 *	17.6 ± 0.9 *	1.1 ± 0.1
Glucose (mg/dL)	NOR	83 ± 2	90 ± 3	85 ± 3	86 ± 3	92 ± 4	92 ± 4	81 ± 1
	LOW	85 ± 2	88 ± 2	86 ± 3	87 ± 3	89 ± 4	91 ± 3	81 ± 1

Values are means ± SE.* *p* < 0.05 vs. Pre.

## Data Availability

Data is contained with in the article.
